# The Apoptotic Effect of HIF-1α Inhibition Combined with Glucose plus Insulin Treatment on Gastric Cancer under Hypoxic Conditions

**DOI:** 10.1371/journal.pone.0137257

**Published:** 2015-09-04

**Authors:** Tomokazu Tanaka, Yoshihiko Kitajima, Shuusuke Miyake, Kazuyoshi Yanagihara, Hiromitsu Hara, Aki Nishijima-Matsunobu, Koichi Baba, Masaaki Shida, Kota Wakiyama, Jun Nakamura, Hirokazu Noshiro

**Affiliations:** 1 Department of Surgery, Saga University Faculty of Medicine, Nabeshima, Saga, Japan; 2 Department of Surgery, NHO Higashisaga Hospital, Harakoga, Miyaki-town, Miyaki-Gun, Saga, Japan; 3 Division of Pathology, Research Center of Innovative Oncology, National Cancer Center Hospital East, Kashiwanoha, Kashiwa, Chiba, Japan; 4 Department of Immunology, Kagoshima University Graduate School of Medical and Dental Sciences, Sakuragaoka, Kagoshima, Japan; 5 Department of Molecular and Tumor Pathology, Akita University Graduate School of Medicine, Hondo, Akita, Japan; University of Oxford, UNITED KINGDOM

## Abstract

Gastric cancer grows under a hypoxic environment. HIF-1α is known to play an important role in controlling the production of reactive oxygen species (ROS) in the mitochondria under hypoxic conditions. We previously established HIF-1α knockdown (KD) cells and control (SC) cells in the 58As9 gastric cancer cell line. In this study, we revealed that KD cells, but not SC cells, induced apoptosis under conditions of hypoxia (1% O_2_) due to excessive production of ROS. A quantitative RT-PCR analysis demonstrated that the expressions of ten genes, which are involved in the control mechanisms of ROS (including the Warburg effect, mitophagy, electron transport chain [ETC] modification and ROS scavenging), were regulated by HIF-1α. Moreover, the promotion of glucose uptake by glucose plus insulin (GI) treatment enhanced the apoptotic effect, which was accompanied by further ROS production in hypoxic KD cells. A Western blot analysis showed that the membranous expression of GLUT1 in KD cells was elevated by glucose and/or insulin treatments, indicating that the GI-induced glucose uptake is mediated by the increased translocation of GLUT1 on the cell membrane. Finally, the anti-tumor effect of HIF-1α knockdown (KD) plus GI was evaluated using a tumor xenograft model, where a hypoxic environment naturally exists. As a result, the GI treatment strongly inhibited the growth of the KD tumors whereby cell apoptosis was highly induced in comparison to the control treatment. In contrast, the growth of the SC tumors expressing HIF-1α was not affected by the GI treatment. Taken together, the results suggest that HIF-1α inhibition plus GI may be an ideal therapy, because the apoptosis due to the destruction of ROS homeostasis is specifically induced in gastric cancer that grows under a hypoxic environment, but not in the normal tissue under the aerobic conditions.

## Introduction

The hypoxic environment is substantial in solid tumors where it accelerates their malignant behaviors [1–4]. Like other solid tumors, gastric carcinoma is known to involve extensive areas of hypoxia within the tumor [5–7]. Hypoxic conditions induce several biological events such as angiogenesis, local invasion, metastatic spread, radio- or chemoresistance and altered energy metabolism in many carcinomas, leading to a poor prognosis in patients [2–4].

The transcription factor hypoxia-inducible factor 1 (HIF-1) is the principal mediator of the cellular adaptation to hypoxia [8–10]. HIF-1 is a heterodimeric protein consisting of a constitutively expressed β-subunit (HIF-1β) and a hypoxia-inducible α (HIF-1α) subunit [8–10]. The HIF-1α subunit is degraded through the ubiquitin-proteasome pathway under normoxia. In contrast, under hypoxia, HIF-1α is stabilized and dimerizes with HIF-1β interacting with CBP/p300, which then binds to the hypoxia response element (HRE) on the promoter region of hundreds of target genes [11–16]. These previous reports have led to the recognition of HIF-1α as a central regulator in the pathogenesis of solid cancer.

Reactive oxygen species (ROS), such as superoxide anion (O_2_^-^), hydrogen peroxide (H_2_O_2_), and hydroxyl radical (HO•), consist of radical and non-radical oxygen species formed by the partial reduction of oxygen. Intracellular ROS are mainly generated in the mitochondria by oxidative phosphorylation (OXPHOS), a process performed by the electron transport chain (ETC) [17]. When ROS overwhelm the cellular antioxidant defense system, oxidative stress occurs. Excessive oxidative stress causes the ROS-mediated damage of nucleic acids, proteins, and lipids and leads to cell death [17, 18].

HIF-1α has been reported to control ROS production under hypoxic conditions through multiple mechanisms including the conversion of energy metabolism from OXPHOS to glycolysis, which is referred to as the Warburg effect [19–23], the induction of mitochondrial selective autophagy (designated as mitophagy) [24, 25], ETC modification by a subunit switch in cytochrome c oxidase (COX) [26] and ROS scavengers [27]. In the metabolic pathway of the Warburg effect, HIF-1α first activates the transcription of *GLUT1* to increase the glucose uptake in cells. Glucose is then metabolized to pyruvate by the actions of glycolytic enzyme members, which are known targets for HIF-1α [28, 29]. Under aerobic conditions, pyruvate is converted to acetyl-CoA (AcCoA) by pyruvate dehydrogenase (PDH) for entry into the tricarboxylic acid (TCA) cycle. Conversely, in cancer cells exposed to hypoxia, pyruvate is shunted away from the mitochondria, whereby HIF-1α upregulates the expression of PDK1 to inhibit the PDH activity. Thereafter, LDHA alternatively converts pyruvate to lactate and MCT4 transports the lactate out of the cell. These genes are also regulated by HIF-1α [16, 30, 31]. Hypoxia induces mitophagy to prevent excessive ROS production by which the damaged mitochondria are eliminated via lysosomal digestion [24, 25]. Recent studies have demonstrated that HIF-1α activates the transcriptions of the genes encoding BNIP3 and BNIP3L, essential factors in the mitophagy process [32]. Another study reported that HIF-1α regulates the COX4 subunit switching by activating the transcription of the ETC-related genes COX4-2 and LON, a mitochondrial protease that is required for COX4-1 degradation under hypoxia [26]. The COX4 subunit switch was reported as an important step in ROS homeostasis, due to its role in optimizing the efficiency of respiration under hypoxia [26]. The ROS scavenger MnSOD is known to convert superoxide radicals to hydrogen peroxide. A previous study has reported that MnSOD is upregulated under hypoxia, although whether this upregulation is mediated by HIF-1α has not yet been shown [27]. Recently, another report demonstrated the interesting finding that the embryonic fibroblasts (MEFs) of hypoxic HIF-1α-null mice died due to excess ROS production, while the MEFs were rescued by treatment with the antioxidant N acetyl-L-cysteine (NAC) [33]. Taken together, these reports indicate that HIF-1α plays a central role in organizing the mitochondrial ROS production in living cells under hypoxia.

In this study, we aimed to establish a therapeutic model demonstrating that hypoxia-induced apoptosis via the overproduction of ROS can be introduced to HIF-1α deficient gastric cancer cells. We initially determined whether hypoxia induces cell death by the excessive ROS production in the HIF-1α knockdown (KD) cells. Thereafter, we addressed the hypothesis that the introduction of high glucose levels by the treatment of KD cells with insulin may enhance the apoptotic effect. Finally, using a tumor xenograft model, we proposed that HIF-1α inhibition combined with glucose plus insulin (GI) treatment may be a potential therapy for gastric cancer.

## Materials and Methods

### Cell culture conditions and reagents

The gastric cancer cell line 58As9 was kindly provided by Dr. K. Yanagihara (National Cancer Center Hospital East, Chiba, Japan) on December in 2009. The 58As9 cell line was originally established from the scirrhous gastric carcinoma-derived cell line HSC-58 [34]. This cell line was then further authenticated on February 24^th^, 2015 by the JCRB Cell Bank (Osaka, Japan). Another gastric cancer cell line, MKN74 was purchased from Cell Bank, RIKEN Bio Resource Center (Tsukuba, Japan). In the present study, we used stable HIF-1α knockdown cells KD and 74-KD, which were established by the transfection of the siRNA plasmid harboring the RNAi sequences into the 58As9 and MKN74 cells as described previously [7, 35]. The sequences of siRNA targeting HIF-1α and control scrambled siRNA were designed as follows: HIF-1α siRNA for KD or 74-KD (5’-CCA CAT TCA CGT ATA TGA T-3’) and scramble siRNA for SC or 74-SC (5’-TCT TAA TCG CGT ATA AGG C-3’). The cells were cultured in RPMI-1640 medium (Sigma-Aldrich, Inc., St. Louis, MO, USA) supplemented with 10% heat-inactivated fetal bovine serum (FBS) and 100 μg/mL kanamycin (Meiji, Tokyo, Japan) and incubated at 37°C in a humidified atmosphere. The cells were cultured under either normoxic conditions (20% O_2_ and 5% CO_2_ in air) or hypoxic conditions (1% O_2_, 5% CO_2_ and 94% N_2_) in a hypoxic chamber (ASTEC, Fukuoka, Japan) and then treated with NAC (Sigma-Aldrich) and insulin (Wako, Osaka, Japan) at a final concentration of 5 mM and 500 ng/ml, respectively. The concentration of the high glucose medium was prepared by the addition of 45% D-(+)-Glucose solution (Sigma-Aldrich) and the final concentration was determined to be 10 g/L, which is 5 times higher than that in normal RPMI-1640 medium.

### Cell viability assay

The cell viability under normoxia or hypoxia was assessed by trypan blue dye exclusion assays. For the evaluation of drug treatment effects, including NAC, high glucose and/or insulin on the cell viability, 1×10^5^ cells were seeded onto 6 cm culture dishes. The cells were treated with various drugs at the indicated concentrations and cultured under normoxia or hypoxia for 24 h to 96 h. At the end of the incubation, the floating and adherent cells were collected and pelleted by centrifugation (3000 rpm, 5 min). The cells were resuspended in 90 μL of the complete medium, mixed with 10 μL of 0.4% trypan blue solution and counted using a hemocytometer under a microscope. The cell death rate was determined as the ratio of the number of dead cells/the total cell number. All experiments were performed in triplicate and independently repeated at least three times.

### Western blot analysis

Whole cell lysates from cultured cells and the xenograft tumors in mice were prepared using lysis buffer composed of 150 mmol/L NaCl, 50 mmol/L Tris-HCl (pH 7.6), 0.5% Triton X-100, and a protease inhibitor cocktail mix (Roche, Mannheim, Germany). Cell lysates from the cytosolic fraction and cell membrane fraction were prepared using a Cytochrome c Releasing Apoptosis Assay Kit and a Plasma Membrane Protein Extraction Kit (BioVision Inc., Milpitas, CA, USA) according to the manufacturer’s instructions. The Western blot analysis was performed as previously described [7]. Aliquots containing 30 μg of protein were electrophoretically separated in 4–12% Bis-Tris Gel (Invitrogen) and transferred onto an Amersham Hybond-ECL membrane (GE Healthcare, Buckinghamshire, UK) in a transfer buffer. After blocking with 5% skin milk for 30 min, the membrane was incubated with primary antibodies overnight at 4°C. The following primary antibodies were used: anti-HIF-1α (1:1000 dilution, Abcam, Cambridge, UK), anti-cleaved caspase 3 (1:1000, Cell Signaling Technology, Danvers, MA), anti-cytochrome c (1:500 dilution, BioVision), anti-GLUT1 (1:100,000 dilution, Abcam), and anti–β-actin (1:10,000 dilution; Sigma-Aldrich, Inc.). Following incubation with the corresponding secondary antibodies, the signals were developed using an Amersham ECL Plus Western Blotting Detection System (GE Healthcare).

### Detection of intracellular ROS by flow cytometry

Intracellular ROS values were evaluated using a Total ROS Detection Kit (Enzo Life Sciences, Inc., Farmingdale, NY, USA) according to the manufacturer’s instructions. In brief, KD, SC, cells were cultured under conditions of either normoxia or hypoxia with or without drug treatments (i.e., NAC, high glucose and/or insulin) for 24 h, 48 h and 72 h. 74-KD and 74-SC were also cultured under conditions of either normoxia or hypoxia for 24, 48, and 72 hours without any drug treatment. The cells were washed and re-suspended in the ROS detection solution. ROS fluorescence was detected by the FACS Calibur flow cytometer (Becton-Dickinson, San Jose, CA) and analyzed by the Cell Quest software program. All experiments were performed in triplicate. The mean fluorescence of ROS production was determined automatically and presented as the GEO mean.

### Total RNA extraction and quantitative RT-PCR

Total RNA was extracted from cell lines using an Isogen RNA extraction kit (Nippon Gene, Osaka, Japan). One μg of RNA was converted into cDNA using a ReverTra Ace (Toyobo) reverse transcription reaction kit. The cDNA was used as a template for the PCR. A real-time quantitative RT-PCR (RT-qPCR) was performed by means of the Light Cycler instrument system (Roche Diagnostics GmbH, Mannheim, Germany) using a Light-Cycler-FastStart DNA Master SYBR Green I kit (Roche). The ten genes that were analyzed by the RT-qPCR were as follows: glucose transporter 1 (*GLUT1*), aldolase C (*ALDOC*), pyruvate dehydrogenase kinase 1 (*PDK1*), lactate dehydrogenase A (*LDHA*) and monocarboxylate transporter 4 (*MCT4*), Bcl-2/adenovirus EIB 19-kDa interacting protein 3 (*BNIP3*), BINP3 like (*BNIP3L*), mitochondrial manganese superoxide dismutase (*MnSOD*), a mitochondrial protease *LON* and cytochrome oxidase subunit 4–2 (*Cox4- 2*). The primers were designed according to the reported cDNA sequences (GenBank, Bethesda, MD) (Table 1). After performing a denaturation step at 95°C for 3 min, PCR amplification was conducted with 50 cycles of 15 s of denaturation at 95°C, 5 s of annealing at 60°C and 10 s of extension at 72°C. The quantitative values were normalized to the β-actin (*ACTB*) expression (Table 1). All experiments were performed in triplicate and independently repeated at least three times.

**Table 1:** Primer sequences for ten genes involved in regulating ROS production.

Genes	Forward primer	Reverse primer
***GLUT1***	5'-ACT GGG CAA GTC CTT TGA GAT-3'	5'-GTC CTT GTT GCC CAT GAT GGA-3'
***ALDOC***	5'-AAA TTG GGG TGG AAA ACA CA-3'	5'-AGA AAA TGA CGC CTC CAA TG-3'
***PDK1***	5'-GGT TAC GGG ACA GAT GCA GT-3'	5'-CGT GGT TGG TGT TGT AAT GC-3'
***LDHA***	5'-GGC CTG TGC CAT CAG TAT CT-3'	5'-CTT TCT CCC TCT TGC TGA CG-3'
***MCT4***	5'-TTG GGT TTG GCA CTC AAC TT-3'	5'-GAA GAC AGG GCT ACC TGC TG-3'
***BNIP3***	5'-ACC CTC AGC ATG AGG AAC AC-3'	5'-ACC CTC AGC ATG AGG AAC AC-3'
***BNIP3L***	5'-GAT GTG GAA ATG CAC ACC AG-3'	5'-TAC CCA GTC CGC ACT TTT CT-3'
***LON***	5'-CGG GAA GAT CAT CCA GTG TT-3'	5'-ACG TCC AGG TAG TGG TCC AG-3'
***COX4-2***	5'-GCT ATG CCC AGC GCT ACT AC-3'	5'-CAT CTC CGC AAA GGT CTC AT-3'
***MnSOD***	5'-CTG GAC AAA CCT CAG CCC TA-3'	5'-CTG ATT TGG ACA AGC AGC AA-3'
***ACTB***	5'-ACT CTT CCA GCC TTC CTT CC-3'	5'-GAC AGC ACT GTG TTG GCG TA-3'

### Glucose uptake assay

The glucose uptake in cultured cells was determined using a 2-Deoxyglucose (2DG) Uptake Measurement Kit (COSMO BIO Co. Ltd., Tokyo, Japan). Briefly, the cells were cultured under a serum-starved condition for 6 h, followed by the further cultivation for 18 h in regular medium supplemented with 10% FBS. The cells were incubated for 24 h under normoxia or hypoxia. Thereafter, the cells were treated with or without 500 ng/ml insulin for 18 min. Finally, the cells were treated with 2DG for 20 min and subjected to the measurement of the 2DG uptake according to the manufacturer’s instructions. All experiments were performed in triplicate and the mean values were calculated.

### Animal studies

The animal protocols were approved by the Institutional Animal Care and Use Committees of Saga University (protocol 24-008-0) and conformed to the ARRIVE guidelines for the use of animals in research. Female 4-week-old athymic BALB/cA Jcl mice (nu/nu) were obtained from Nihon Crea Co. (Osaka, Japan). Animals were kept under specific-pathogen-free conditions. They were given sterile food and autoclaved water with a 12-h light-dark cycle. Mice were acclimated to the environment for 7 days before the experiments. KD or SC cells (3×10^6^) were injected subcutaneously into the backs of the mice (n = 9 for each cell line). Ten days after the subcutaneous inoculation, the xenografts of both cells became palpable. Nine mice bearing KD or SC xenografts were then divided into three groups for treatment by glucose (8 g/kg/day, Sigma), glucose plus insulin (GI) (1 unit per 3 g glucose/day, Wako) or phosphate-buffered saline (PBS) as the control treatment. Each of these drugs were intraperitoneally administered into three mice (six tumors total) every 24 h from day 1 to day 11. During this period, the tumors were measured in 2 perpendicular dimensions with a caliper every four days. The tumor size (*T*) was evaluated as the maximum cut area and determined by the following formula: *T* = π/4 × *a* × *b*, where *a* is the shorter axis (mm) and *b* is the longer axis (mm). The mice were sacrificed 12 days after the drug treatments and the tumors were harvested for the subsequent experiment.

### Cleaved caspase 3 immunohistochemistry and the assessment of apoptosis *in vivo*

The frozen tumors were embedded with Tissue-Tek O.C.T. Compound. These blocks were cut into 4-μm-thick sections. For antigen retrieval, the slides were heated in Tris-EDTA buffer (pH 9.0) in a microwave (500 watt) for 5 min. The sections were then incubated with anti-cleaved caspase 3 (1:200, Cell Signaling Technology) for 2 h at room temperature, and the DAKO Envision+ System (Dako Cytomation, Glostrup, Denmark) was used as the secondary antibody. The signals were visualized with diaminobenzidine tetrahydrochloride (0.02%). For the assessment of apoptosis, cleaved caspase 3-positive cells with brown colored nuclei were counted in five fields at 400x magnification and the mean was calculated. The immunohistochemical expression of cleaved caspase 3 was blindly reviewed and assessed by a certified pathologist (Dr. A.N.).

### Statistical analysis

The data were analyzed by an ANOVA using the Prism 5 software package (GraphPad Software, La Jolla, CA). For the comparison between two groups, the differences in the mean values were evaluated by Student’s *t-*test and the Mann-Whitney *U* test. For the comparison among three or more groups, Bonferroni post hoc tests were performed for a one-way ANOVA. A value of p<0.05 was considered to be statistically significant. All data are expressed as the means ± SEM.

## Results

### HIF-1α knockdown induced apoptotic cell death under hypoxia in 58As9 gastric cancer cells

**Fig 1:**
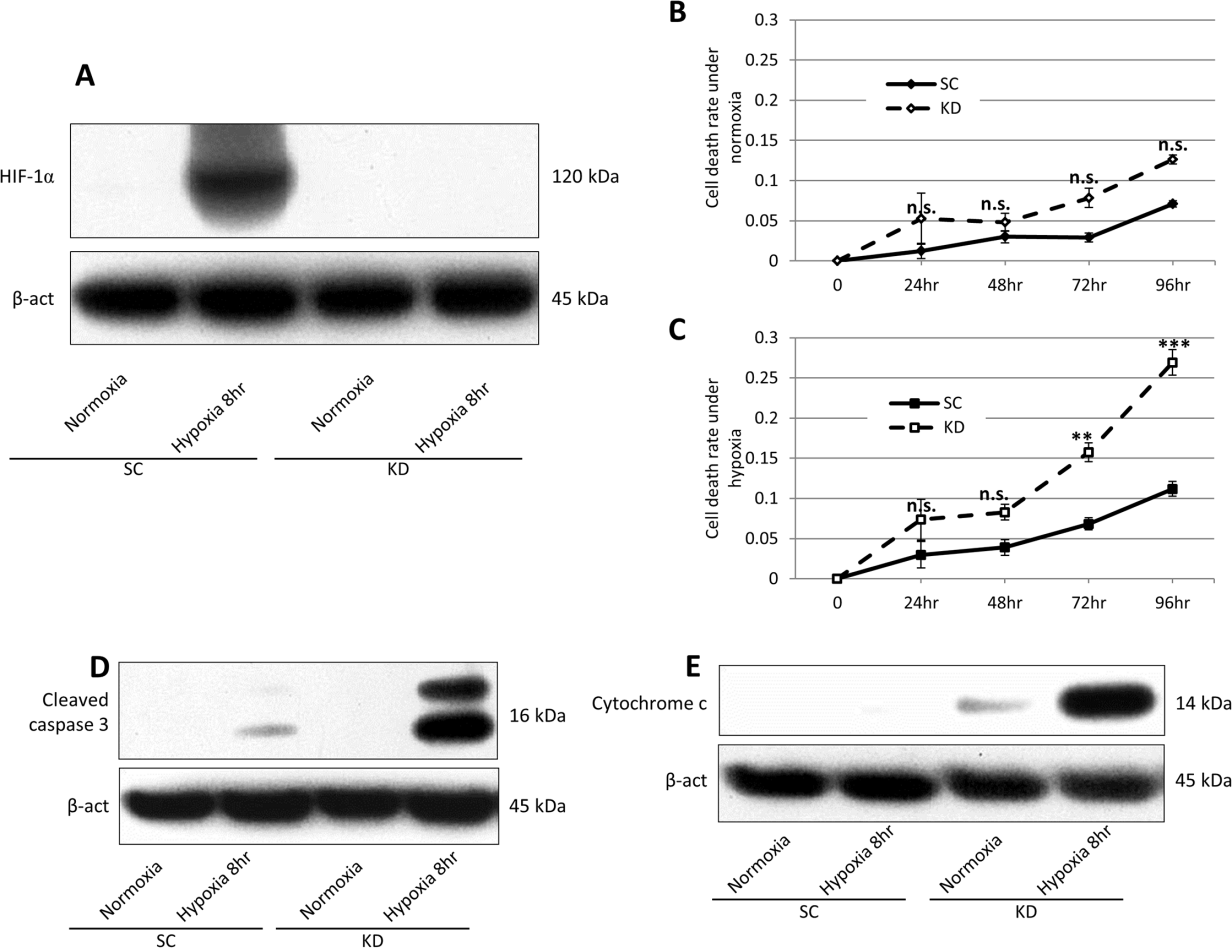
Hypoxia-induced apoptosis in HIF-1α knockdown KD cells. (A) The Western blot analysis of the HIF-1α expression was performed in the SC or KD cells under normoxia (20% O_2_) and hypoxia (1% O_2_). The internal marker β-actin (β-act) was equally expressed in all cells. (B), (C) The cell death rate was assessed using SC cells or KD cells under normoxia (B) and hypoxia (C) for 0 h to 96 h. The death rates were compared between SC cells and KD cells. n.s.: not significant, *: p<0.05, **: p<0.01, ***: p<0.001. (D), (E) A Western blot analysis of (D) cleaved caspase 3 and (E) cytosolic cytochrome c in the SC or KD cells under normoxia and hypoxia for 8 h.

The HIF-1α expression was assessed in stable HIF-1α knockdown (KD) cells and SC (as the control cell line) cells. A Western blot analysis showed that the HIF-1α expression was completely knocked down in the KD cells after 8 hours under hypoxia compared with the SC cells (Fig 1A). The cell death rate was estimated after 24 h to 96 hours under normoxia and hypoxia. The death rate was higher in the KD cells than in the SC cells under normoxia for 24 to 96 hours, however the differences were not statistically significant (Fig 1B). In contrast, the cell death rate in the KD cells was strongly increased under hypoxia and significantly higher than that observed in the SC cells at 72 and 96 hours (Fig 1C). The Western blot analysis demonstrated that cleaved caspase 3 as well as cytosolic cytochrome c were elevated in the KD cells, but not in the SC cells under hypoxia for 8 hours (Fig 1D and 1E). Hypoxia induced cell death was also confirmed in other HIF-1α knockdown gastric cancer cells 74-KD (S1 Fig). These results indicated that hypoxia strongly induced apoptosis in the HIF-1α knockdown cell line KD.

### Scavenging ROS reversed the apoptotic phenotype observed in HIF-1α knockdown cells

**Fig 2:**
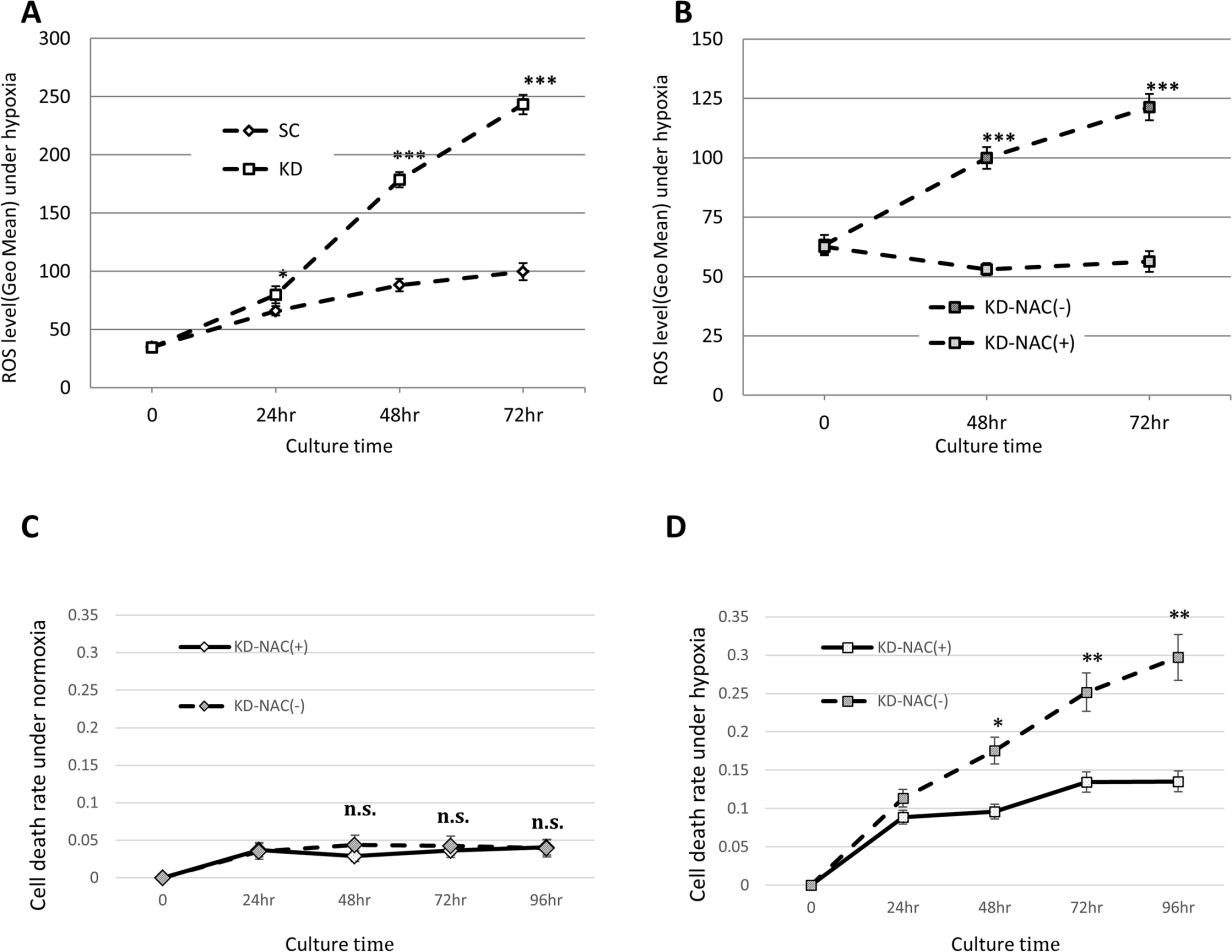
Scavenging ROS reversed the apoptotic phenotype observed in HIF-1α knockdown cells under hypoxia. (A) The ROS levels were estimated using the KD and SC cells under hypoxia for 0 h to 72 h. (B) The ROS level was analyzed in hypoxic KD cells with or without 5 mM NAC treatment for 0 to 72 h. (C) The cell death rate was assessed in KD cells with or without NAC under normoxia (C) and hypoxia (D) for 0 h to 96 h. KD-NAC(-): NAC-untreated KD cells, KD-NAC(+): NAC-treated KD cells. *: p<0.05, ***: p<0.001.

The intracellular ROS level was estimated and compared among the KD and SC cells. The ROS level increased in a time-dependent manner in the KD cells under hypoxia, while the level was faintly elevated in the SC cells (Fig 2A). The ROS level in the KD cells was significantly higher under hypoxia for 24 to 72 hours than that in the SC cells (Fig 2A). The ROS levels were also assessed in the 74-SC cells and 74-KD cells (S2 Fig). The ROS levels did not differ between the 74-SC and 74-KD cells under normoxia (S2A Fig). However, under hypoxia, the ROS levels were significantly higher in the 74-KD cells than in the 74-SC cells at 48 to 72 hours (S2B Fig). NAC, an antioxidant, significantly decreased the ROS level in the KD cells under hypoxia for 48 to 72 hours (Fig 2B). In order to assess whether ROS production induces hypoxia-induced cell death in KD cells, the cell death rate with or without NAC was evaluated in KD cells under normoxia and hypoxia. NAC treatment did not affect the rate of cell death in the KD cells under normoxia (Fig 2C). In contrast, NAC treatment significantly reduced the cell death in the KD cells under hypoxia for 48 to 96 hours (Fig 2D).

### HIF-1α knockdown reduced the hypoxic induction of various genes involved in the control of ROS production

**Fig 3:**
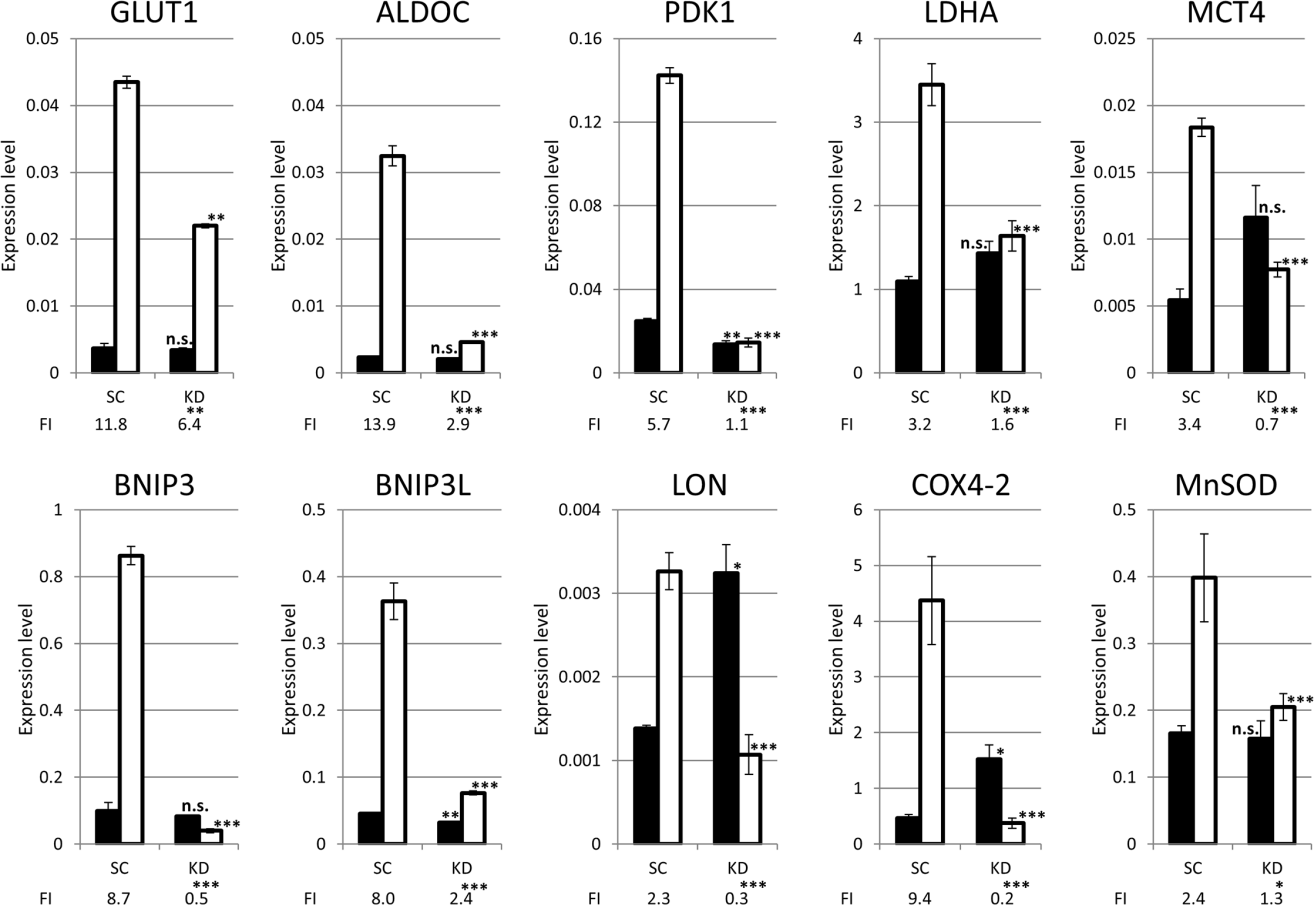
The RT-qPCR analysis of ten genes in SC or KD cells under normoxia and hypoxia. The mRNA expression of GLUT1, ALDOC, PDK1, LDHA, MCT4, BNIP3, BNIP3L, LON, COX4-2 and MnSOD was quantitatively assessed under normoxia (black bars) and hypoxia (white bars) for 24 hours. The expression levels are presented in the graph. The fold induction (FI) was estimated as the expression ratio of hypoxia/normoxia and is presented on the bottom of the graphs. The expression levels of ten genes in the KD cells were compared with the expression levels in the SC cells under both normoxia and hypoxia. ns: not significant, *: p<0.05, **: p<0.01, ***: p<0.001.

To investigate the hypoxia-induced ROS accumulation in the HIF-1α knockdown cells, the mRNA expression of ten genes, which are involved in the ROS control mechanism (*GLUT1*, *ALDOC*, *PDK1*, *LDHA*, *MCT4*, *BNIP3*, *BNIP3L*, *LON*, *COX4-2* and *MnSOD*) were analyzed using a RT-qPCR. The hypoxic induction of the gene expression was assessed by the fold induction (FI). The FIs in the ten genes were significantly lower in the KD cells than in the SC cells (Fig 3). Furthermore, when restricted to hypoxic conditions, the expression levels of all ten genes were significantly lower in the KD cells than the SC cells. These results showed that HIF-1α knockdown markedly decreased the hypoxia-induced expression of the ten genes. On the other hand, under normoxia, the expression of PDK1 and BNIP3L was significantly lower in the KD cells than in the SC cells. Conversely, the expression levels of LON and COX4-2 were significantly higher in the KD cells in comparison to the SC cells.

### Glucose and insulin treatments enhanced the apoptotic cell death in KD cells under hypoxia

**Fig 4:**
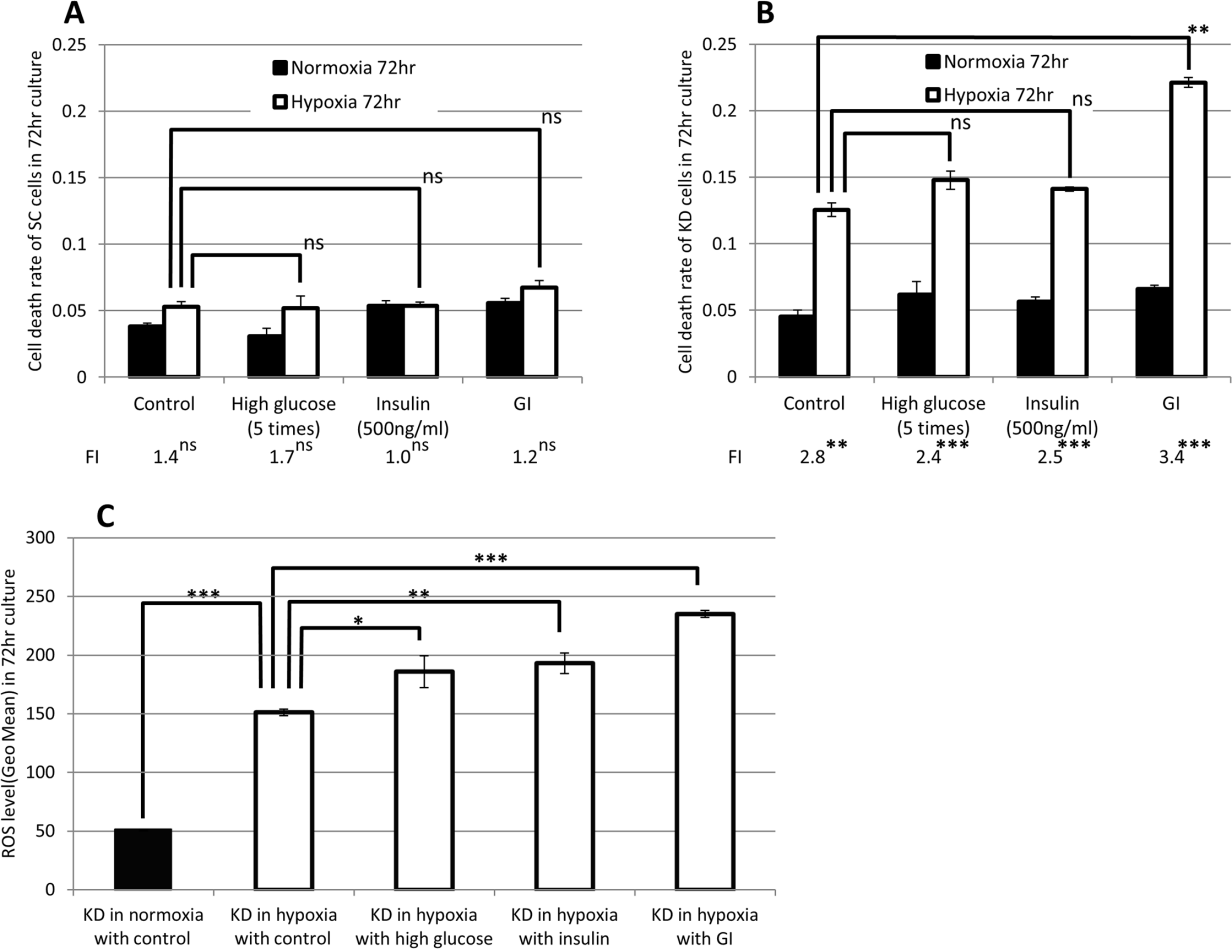
The effect of GI treatment on cell death and ROS production. The FI of the cell death rate was determined as the death ratio of hypoxia/normoxia. The cell death rate in PBS-treated (control), high glucose and/or insulin-treated SC cells (A) and KD cells (B) is plotted. The FI value is presented on the bottom. The cell death rate under hypoxic conditions in the control treatment was compared with that in high glucose, insulin and GI treatment. (C) The intracellular ROS level in the control-treated, high glucose and/or insulin-treated KD cells are plotted on the graph. The ROS levels in the KD cells treated with the control treatment were compared between normoxia (black bars) and hypoxia (white bars). The ROS level in the hypoxic KD cells with control treatment was further compared with that with high glucose, insulin and GI treatment. ns: not significant, *: p<0.05, **: p<0.01, ***: p<0.001.

We next investigated whether the promotion of the glucose uptake affects the hypoxia-induced apoptosis in KD cells. The cell viability was assessed in the KD and SC cells following treatments with control (PBS), high glucose, insulin or high glucose plus insulin (GI). Hypoxia-induced cell death was estimated by the FI. The cell death rate under hypoxia was compared between the control treatment and the other treatments in both cell lines (Fig 4). In the SC cells, no significant differences in the FI and cell death rate under hypoxia were observed among any of the treatments (Fig 4A). In the KD cells, the FI was significantly increased by hypoxia in all treatments. In particular, GI treatment yielded the highest FI among all treatments (Fig 4B). The cell death rate under hypoxia was significantly higher in the GI-treated cells than in the control-treated cells (Fig 4B). To investigate whether the treatments affected the ROS production, the ROS level was analyzed in the KD cells. In comparison to normoxic conditions, the ROS level was significantly elevated in the KD cells under hypoxic conditions (Fig 4C). Under hypoxia, the ROS level in KD cells was significantly increased by high glucose, insulin and GI treatment in comparison to control treatment. The highest ROS level was positively observed in the GI-treated KD cells (Fig 4C).

### Assessment of the glucose uptake after insulin treatment

**Fig 5:**
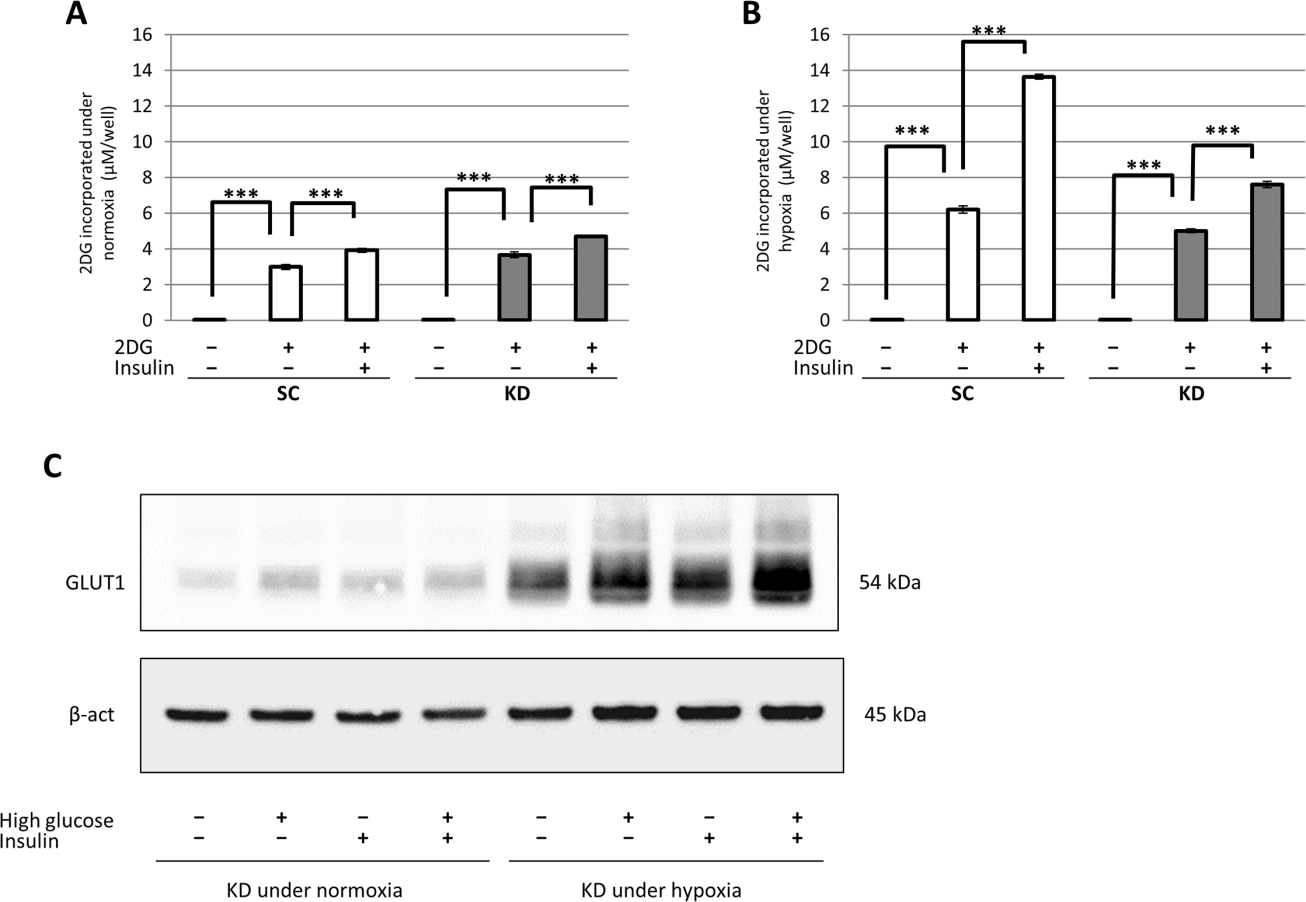
The examination of glucose uptake in SC and KD cells with or without insulin treatment. (A), (B) The 2DG uptake level in SC cells or KD cells with or without insulin treatment was evaluated under normoxia (A) and hypoxia (B). (C) The Western blot analysis of membranous GLUT1 (54 kDa) expression in the KD cells with control, high glucose and/or insulin treatments under both normoxia and hypoxia as indicated. ***: p<0.001.

The glucose uptake ability was analyzed in a 2DG incorporation study. In the SC and KD cells under normoxia, the 2DG incorporation was significantly elevated by the 2DG treatment in comparison to untreated cells. The 2DG incorporation was further increased by the additional insulin treatment in both cells (Fig 5A). In comparison to normoxia, hypoxia more strongly stimulated the 2DG uptake in the SC cells, with or without insulin (Fig 5B). Similar findings were observed in the KD cells under hypoxia (Fig 5B). However, under hypoxia, less 2DG was incorporated into the KD cells than in the SC cells, with or without the additional insulin treatment (Fig 5B). To assess the mechanism of insulin-dependent glucose uptake, the membranous expression of GLUT1 was analyzed in the KD cells under normoxia and hypoxia. Under normoxia, the membranous GLUT1 expression was elevated with high glucose and/or insulin treatment in comparison to that with no treatment (Fig 5C). Compared to that observed under normoxia, under hypoxia, the membranous GLUT1 expression was elevated in all treatments. Furthermore, the expression was increased by high glucose and/or insulin treatment, compared with that by no treatment (Fig 5C). In particular, the membranous GLUT1 expression was most strongly increased by high glucose and insulin (GI) treatment in the hypoxic KD cells (Fig 5C). On the other hand, the expression of another GLUT family, GLUT3, was faintly observed in the KD cells, and this finding was not altered among these various treatments (data not shown). In this study, GLUT2 and GLUT4 were not expressed in the KD cells.

### HIF-1α knockdown plus GI treatment strongly suppressed the growth of tumor xenografts in nude mice

**Fig 6:**
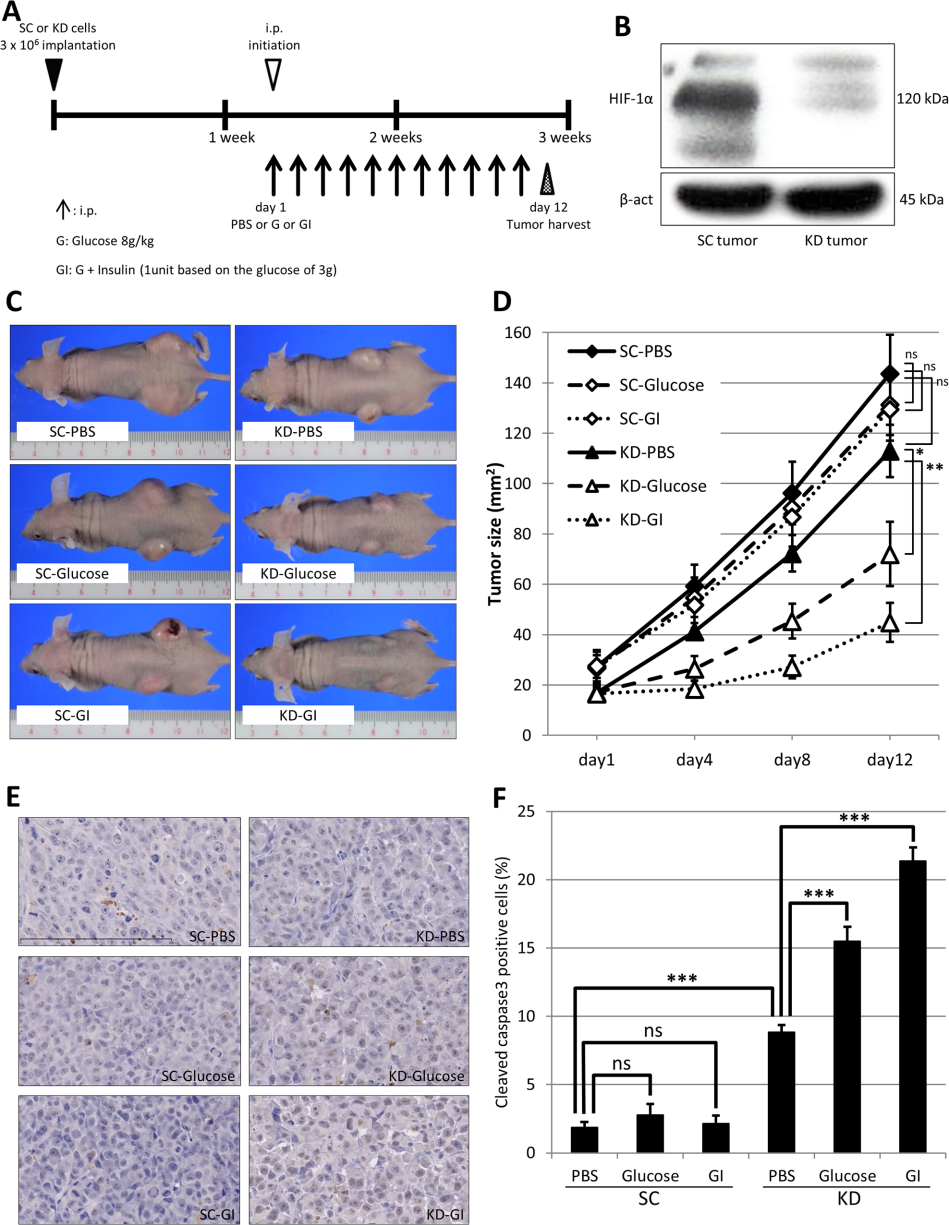
The *in vivo* effect of HIF-1αknockdown plus GI treatment on tumor xenografts. (A) The experimental schedule of glucose (G) or glucose plus insulin (GI) treatment on the tumor xenografts. The initial intraperitoneal (i.p) injection with control PBS or Glucose, GI is indicated by the empty triangle. The i.p injections are indicated by arrows. The tumors harvested on day 12 are indicated by the reticulated triangle. (B) The Western blot analysis of HIF-1α in the xenografts of the SC cells (SC tumor) and KD cells (KD tumor). (C) Representative images of the SC or KD tumors treated with PBS, Glucose or GI. The treated tumors were designated as SC-PBS, SC-Glucose, SC-GI, KD-PBS, KD-Glucose and KD-GI. (D) The size of the 6 tumors on day 1 to day 12 was plotted on the graph as indicated. (E) The immunohistochemical analysis of cleaved caspase 3 was performed using six tumors of SC-PBS, SC-Glucose, SC-GI, KD-PBS, KD-Glucose and KD-GI. (F) The mean positive rate of cleaved caspase 3 in 6 tumors was estimated and presented in the graph. ns: not significant, *: p<0.05, **: p<0.01, ***: p<0.001.

Finally, we determined the *in vivo* effect of the GI treatment on KD and SC tumor xenografts. Fig 6A demonstrates the experimental design of the xenograft murine model. Ten days after the subcutaneous inoculation of SC or KD cells, xenografts were grown on the backs of nude mice. At this point, a Western blot analysis confirmed the HIF-1α expression in the SC tumors, but not in the KD tumors (Fig 6B). Thereafter, three drugs, consisting of PBS, glucose or GI, were intraperitoneally injected into nude mice bearing an SC or KD tumor (daily from day 1 to day 11). The representative images of the tumor-bearing mice that were treated with PBS (SC-PBS and KD-PBS), glucose (SC-Glucose and KD-Glucose) or GI (SC-GI and KD-GI) are shown in Fig 6C. The KD-Glucose and KD-GI tumors appeared to be smaller than the other tumors. Fig 6D showed the growth curve of the 6 tumors. The sizes of the KD-Glucose and KD-GI tumors were significantly smaller than the KD-PBS tumor on day 12. The KD-GI tumor was the smallest. On the other hand, in the SC mice, there was no significant difference in size of the SC-PBS, SC-Glucose and SC-GI tumors (Fig 6D). An immunohistochemical analysis of cleaved caspase 3 was performed to assess the apoptosis induced by glucose or GI treatment. The positive expression of cleaved caspase3 was frequently observed in the KD-Glucose and KD-GI tumors (Fig 6E). However, all of the KD tumors exhibited some degree of cleaved caspase 3 (Fig 6F). In contrast, there was no significant difference in expression of the cleaved caspase 3 among the SC-PBS, SC-Glucose and SC-GI tumors. In the KD tumors, the positive expression of cleaved caspase3 was significantly higher in the KD-PBS tumor than in the SC-PBS tumor. Moreover, the expression of cleaved caspase3 was significantly higher in the KD-Glucose or KD-GI tumors than in the KD-PBS tumor. The highest expression was observed in the KD-GI tumor.

## Discussion

In the present study, HIF-1α knockdown cells were used to investigate whether lethal ROS production may be induced under hypoxia. The results showed that the apoptotic cell death was induced in KD cells, but not in the control SC cells under hypoxia. Simultaneously, ROS accumulated in the knockdown cell line according to the duration of hypoxia. Hypoxia- induced cell death and ROS accumulation were also observed in the different HIF-1α knockdown 74-KD cells, but not in the control 74-SC cells. Moreover, the NAC treatment reduced the rate of hypoxia-induced cell death in the KD cells. These results indicate the occurrence of hypoxia-induced apoptosis due to excessive ROS accumulation in KD cells and supported a previous study that HIF-1α knockout MEFs died due to excessive ROS production [33].

ROS are mainly generated in the mitochondria by the ETC [17, 19]. It has been reported that the mitochondrial ROS are increased under hypoxic conditions [17, 19]. If hypoxia persists, the induction of HIF-1α leads to adaptive mechanisms to reduce ROS and reestablish redox homeostasis via the upregulation of relevant genes [19]. We thus investigated whether HIF-1α knockdown affected the mRNA expression of ten genes involved in the control of ROS production under hypoxia (*GLUT1*, *ALDOC*, *PDK1*, *LDHA* and *MCT4* relating to the Warburg effect; *BNIP3* and *BNIP3L* relating to mitophagy; *LON* and *COX4-2* relating to the ETC; and *MnSOD*, a ROS scavenger). The integrated analyses of the RT-qPCR series demonstrated that the hypoxia-induced expression of all ten genes was significantly suppressed in the KD cells, whereas, under normoxia, the mRNA expression of LON and COX4-2 was significantly higher in the KD cells than in the SC cells. These results indicated that the lethal accumulation of ROS under hypoxia in the HIF-1α knockdown cells might be due to multiple disruptions of the ROS control mechanism. Therefore, cancer therapy targeting HIF-1α may be effective in the hypoxic region within gastric cancer tissue. The mechanism underlying the upregulated mRNA expression of LON and COX4-2 in the KD cells under normoxia could not be clarified.

**Fig 7:**
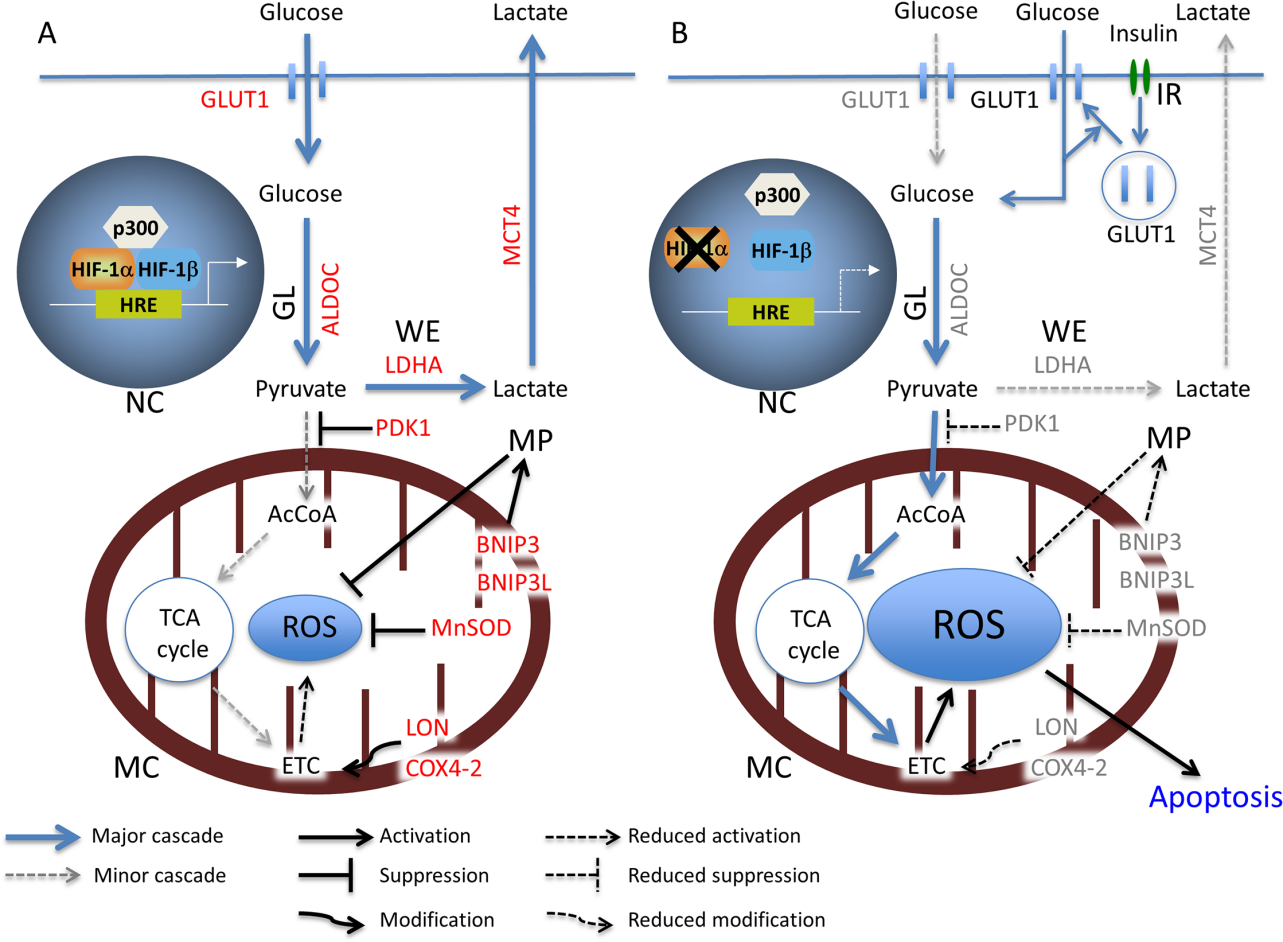
The anti-tumor effect of GI treatment on HIF-1α knockdown KD cells under hypoxic conditions. (A) HIF-1α plays a central role in controlling the ROS level in the SC cells under hypoxia. HIF-1α induces the gene expression of ten specific genes under hypoxia (indicated by red letters). (B) The apoptotic effect induced by HIF-1α knockdown plus GI treatment in the KD cells under hypoxia. The hypoxia-induced expression of the 10 genes is reduced by HIF-1α knockdown (indicated by gray letters). NC: Nucleus, MC: Mitochondria, GL: Glycolysis, WE: Warburg effect, MP: Mitophagy, IR: Insulin receptor.

According to the above findings, we hypothesized that the promotion of the glucose uptake may accelerate hypoxia-induced apoptosis through further ROS production in HIF-1α knockdown KD cells. As expected, the GI treatment enhanced the cell death in hypoxic KD cells, which was accompanied by increased ROS production. In the glucose uptake study, insulin increased the 2DG uptake in both the SC and KD cells. In comparison to that observed under normoxia, hypoxia increased the 2DG uptake in the SC and KD cells treated with or without insulin. The uptake was increased more strongly in the SC than KD cells, suggesting a difference due to the attenuated GLUT1 induction in the hypoxic KD cells. On the other hand, a Western blot analysis showed that the membranous GLUT1 expression was increased with high glucose and/or insulin treatments, compared with control treatment in the normoxic and hypoxic KD cells. In particular, GI treatment most strongly increased the membranous GLUT1 expression in hypoxic KD cells. A previous study reported that insulin promotes glucose transport by stimulating the translocation of GLUT4 from intracellular storage vesicles to the plasma membrane in skeletal muscle cells or adipocytes [36, 37]. Another study has reported the translocation of GLUT1 and GLUT4 on the cell surface by insulin treatmen via a different pathway of signal transduction in mouse NIH3T3-L1 adipocytes [38]. In addition, a previous study has reported that the chronic hyperglycemia also upregulated GLUT1 expression and promoted the K-Ras-induced lung tumorigenesis in mice [39]. In the present study, the Western blot analysis did not demonstrate positive GLUT4 expression in KD cells (data not shown). Therefore, glucose and/or insulin treatments may stimulate GLUT1 translocation to the plasma membrane in KD cells and may have contributed to the elevated 2DG uptake. Moreover, the membranous GLUT1 expression associated with the various treatments was higher under hypoxia than normoxia in the KD cells. In the RT-qPCR analysis, the hypoxic induction of GLUT1 mRNA was preserved in the KD cells, although the degree of induction was reduced in these cells compared with the SC cells. Therefore, the hypoxia-induced expression of GLUT1 in KD cells may also lead to the higher 2DG uptake noted in the KD cells under hypoxia than normoxia with or without insulin treatment. When GI treatment is applied to KD cells under hypoxic conditions, a high amount of glucose may be incorporated into these cells through hypoxia- and GI-induced GLUT1 expression on the cell membrane. As a result, some additional glucose may subsequently be introduced into the glycolysis pathway and converted to AcCoA, even under hypoxic conditions, leading to further ROS production through the ETC, and enhanced apoptosis in hypoxic KD cells. In contrast, the elevated glucose uptake by the GI treatment did not affect the cell viability of SC cells under hypoxia as HIF-1α may control the ROS production at the physiological level. Finally, we analyzed the *in vivo* effect of HIF-1α knockdown plus GI treatment using a tumor xenograft model. A Western blot analysis revealed HIF-1α expression in the SC tumors. These results indicate that spontaneous hypoxia might persist within the tumor xenograft, although an accurate assessment of the extent of tumor hypoxia may be needed using immunostaining for a hypoxia marker, pimonidazole. During the drug treatment, neither the glucose treatment nor GI treatment affected the size of the SC tumors. In contrast, the growth of the KD tumors was inhibited by both the glucose and the GI treatment (in comparison to the PBS treatment). Furthermore, the GI treatment exhibited a stronger inhibition than the glucose treatment. The GI treatment also enhanced apoptosis in the KD tumors more strongly than the control or glucose treatments. In this model, none of the mice died due to the glucose or GI treatment. GI treatment may not exhibit harmful effects on normal tissue at the dose determined in this study. However, using the xenograft model, an estimation of the glucose concentration in the blood may be necessary to assess complications, such as acute hyperglycemia or hypoglycemia due to GI treatment, prior to clinical application. Based on the findings of the present study, a possible mechanism for the anti-tumor effect of GI treatment in KD cells under hypoxia is shown in Fig 7. In the future, instead of HIF-1α knockdown, HIF-1α inhibiting drugs may enable this combined therapy to be clinically feasible. Although small-molecule inhibitors of HIF-1α have been developed, none of these have been clinically approved due to the lack of objective effects and the high toxicity [40–43]. In the present study, KD-PBS still exhibited tumor growth, although higher apoptosis was observed in comparison to the SC tumors. Treatment with a HIF-1α inhibitor alone may not be sufficient to inhibit the tumor growth. In conclusion, HIF-1α inhibition combined with GI treatment may be a promising target for the hypoxic region in gastric cancer, where conventional chemotherapy often fails.

## Supporting Information

S1 Fig Hypoxia-induced cell death in other HIF-1α knockdown cells 74-KD. (A) A Western blot analysis of the HIF-1α expression was performed using 74-SC or 74-KD cells under normoxia (20% O_2_) and hypoxia (1% O_2_). The HIF-1α expression was completely knocked down in the 74-KD cells after eight hours under hypoxia in comparison to that observed in the 74-SC cells. The internal marker β-actin (β-act) was equally expressed in all cells. (B) The cell death rate was not different between the 74-KD cells and 74-SC cells under normoxia for 24 to 96 hours. (C) The cell death rate in the 74-KD cells was significantly higher than that noted in the 74-SC cells under hypoxia for 48 to 96 hours. n.s.: not significant, *: p<0.05, **: p<0.01, ***: p<0.001
(TIF)

S2 Fig Intracellular ROS accumulated in the 74-KD cells under hypoxic conditions. The ROS levels were estimated using 74-SC and 74-KD cells under normoxia (A) and hypoxia (B) for 0 to 72 hours as indicated. n.s.: not significant, *: p<0.05, **: p<0.01, ***: p<0.001
(TIF)

## References

[1] Iyer NV, Kotch LE, Agani F, Leung SW, Laughner E, Wenger RH, et al Cellular and developmental control of O_2_ homeostasis by hypoxia-inducible factor 1 alpha. Genes Dev. 1998; 12: 149–162 9436976 10.1101/gad.12.2.149 PMC316445

[2] Harris AL. Hypoxia- a key regulatory factor in tumour growth. Nat Rev Cancer. 2002; 2: 38–47. 11902584 10.1038/nrc704

[3] Vaupel P, Mayer A, Hockel M. Tumor hypoxia and malignant progression. Methods Enzymol. 2004; 381: 335–354. 15063685 10.1016/S0076-6879(04)81023-1

[4] Lu X, Kang Y. Hypoxia and hypoxia-inducible factors (HIFs): Master regulators of metastasis. Clin Cancer Res. 2010; 16: 5928–5935. 10.1158/1078-0432.CCR-10-1360 20962028 PMC3005023

[5] Rohwer N, Cramer T. HIFs as central regulators of gastric cancer pathogenesis. Cancer Biology & Therapy. 2010; 10: 383–385. 20647752 10.4161/cbt.10.4.12850

[6] Kitajima Y, Miyazaki K. The critical impact of HIF-1α on gastric cancer biology. Cancers (Basel). 2013; 5: 15–26. 24216696 10.3390/cancers5010015 PMC3730315

[7] Nakamura J, Kitajima Y, Kai K, Hashiguchi K, Hiraki M, Noshiro H, et al HIF-1 alpha is an unfavorable determinant of relapse in gastric cancer patients who underwent curative surgery followed by adjuvant 5-FU chemotherapy. Int J Cancer. 2010; 127: 1158–71. 10.1002/ijc.25129 20020496

[8] Majmundar AJ, Wong WJ, Simon MC. Hypoxia inducible factors and the response to hypoxic stress. Mol Cell. 2010; 40: 294–309. 10.1016/j.molcel.2010.09.022 20965423 PMC3143508

[9] Poellinger L, Johnson RS. HIF-1 and hypoxic response: the plot thichens. Curr Opin Genet Dev. 2004; 14: 81–85. 15108809 10.1016/j.gde.2003.12.006

[10] Lu X, Kang Y. Hypoxia and hypoxia-inducible factors (HIFs): master regulators of metastasis. Clin Cancer Res. 2010; 16: 5928–5935. 10.1158/1078-0432.CCR-10-1360 20962028 PMC3005023

[11] Stoeltzing O, McCarty MF, Wey JS, Fan F, Liu W, Belcheva A, et al Role of hypoxia-inducible factor 1α in gastric cancer cell growth, angiogenesis, and vessel maturation. J. Natl. Cancer Inst. 2004; 96: 946–956. 15199114 10.1093/jnci/djh168

[12] Forsythe JA, Jiang BH, Iyer NV, Agani F, Leung SW, Koos RD, et al Activation of vascular endothelial growth factor gene transcription by hypoxia-inducible factor 1. Mol Cell Biol. 1996;16: 4604–46 8756616 10.1128/mcb.16.9.4604 PMC231459

[13] Hara S, Nakashiro K, Klosek SK, Ishikawa T, Shintani S, Hamakawa H. Hypoxia enhances c-Met/HGF receptor expression and signaling by activating HIF-1alpha in human salivary gland cancer cells. Oral Oncol. 2006; 42: 593–598. 16469527 10.1016/j.oraloncology.2005.10.016

[14] Ide T, Kitajima Y, Miyoshi A, Ohtsuka T, Mitsuno M, Ohtaka K, Koga Y, et al Tumor-stromal cell interaction under hypoxia increases the invasiveness of pancreatic cancer cells through the hepatocyte growth factor/c-Met pathway. Int J Cancer. 2006; 119: 2750–2759. 16998831 10.1002/ijc.22178

[15] Comerford KM, Wallace TJ, Karhausen J, Louis NA, Montalto MC, Colgan SP. Hypoxia-inducible factor-1-dependent regulation of the multidrug resistance (MDR1) gene. Cancer Res. 2002; 62: 3387–3394. 12067980

[16] Semenza GL, Jiang BH, Leung SW, Passantino R, Concordet JP, Marie P, et al Hypoxia response elements in the aldolase A, enolase 1, and lactate dehydrogenase A gene promoters contain essential binding sites for hypoxia-inducible factor 1. J Biol Chem. 1996; 271: 32529–32537. 8955077 10.1074/jbc.271.51.32529

[17] Ray PD, Huang BW, Tsuji Y. Reaction oxygen species (ROS) homeostasis and redox regulation in cellular signaling. Cell Signal. 2012; 24: 981–990. 10.1016/j.cellsig.2012.01.008 22286106 PMC3454471

[18] Zou P, Zhang J, Xia Y, Kanchana K, Guo G, Chen W, et al ROS generation mediates the anti-cancer effects of WZ35 via activating JNK and ER stress apoptotic pathways in gastric cancer. Oncotarget. 2015; 6: 5860–5876. 25714022 10.18632/oncotarget.3333 PMC4467407

[19] Semenza GL. Hypoxia-inducible factor 1: regulator of mitochondrial metabolism and mediator of ischemic preconditioning. Biochim Biophys Acta. 2011; 1813: 1263–8. 10.1016/j.bbamcr.2010.08.006 20732359 PMC3010308

[20] Warburg O. On the origin of cancer cells. Science. 1956; 123: 309–314. 13298683 10.1126/science.123.3191.309

[21] Semenza GL. HIF-1: upstream and downstream of cancer metabolism. Curr Opin Genet Dev. 2010; 51: 1–10. 10.1016/j.gde.2009.10.009 PMC2822127 19942427

[22] Cairns RA, Harris IS, Mak TW. Regulation of cancer cell metabolism. Nat Rev Cancer. 2011; 11: 85–95. 10.1038/nrc2981 21258394

[23] Vander Heiden MG, Cantley LC, Thompson CB. Understanding the Warburg effect: the metabolic requirements of cell proliferation. Science 2009; 324: 1029–1033. Journal of Endocrinology. 2004; 183: 145–154. 10.1126/science.1160809 19460998 PMC2849637

[24] Zhang H, Bosch-Marce M, Shimoda LA, Tan YS, Baek JH, Wesley JB, et al Mitochondrial autophagy is an HIF-1-dependent adaptive metabolic response to hypoxia. J Biol Chem. 2008; 283: 10892–10903. 10.1074/jbc.M800102200 18281291 PMC2447655

[25] Ding WX, Yin XM. Mitophagy: mechanisms, pathophysiological roles, and analysis. Biol Chem. 2012; 393: 547–564. 10.1515/hsz-2012-0119 22944659 PMC3630798

[26] Fukuda R, Zhang H, Kim JW, Shimoda L, Dang CV, Semenza GL. HIF-1 regulates cytochrome oxidase subunits to optimize efficiency of respiration in hypoxic cells. Cell. 2007; 129: 111–122. 17418790 10.1016/j.cell.2007.01.047

[27] Ohman T, Parish G, Jackson RM. Hypoxic modulation of manganese superoxide dismutase promoter activity and gene expression in lung epithelial cells. Am. J. Respir. Cell Mol. Biol. 1999; 21: 119–127, 10385600 10.1165/ajrcmb.21.1.3521

[28] Hayashi M, Sakata M, Takeda T, Yamamoto T, Okamoto Y, Sawada K, et al Induction of glucose transporter 1 expression through hypoxia-inducible factor 1α under hypoxic conditions in trophoblast-derived cells. J Endocrinol. 2004;183: 145–54. 15525582 10.1677/joe.1.05599

[29] Jean JC, Rich CB, Joyce-Brady M. Hypoxia results in an HIF-1-dependent induction of brain-specific aldolase C in lung epitjelial cells. Am J Physiol Lung Cell Mol Physiol. 2006; 291: L950 –L956. 16798780 10.1152/ajplung.00087.2006

[30] Kim JW, Gao P, Liu YC, Semenza GL, Dang CV. HIF-1-mediated expression of pyruvate dehydrogenase kinase: a metabolic switch required for cellular adaptation to hypoxia. Cell Metab. 2006; 3: 177–185. 16517405 10.1016/j.cmet.2006.02.002

[31] Ullah MS, Davies AJ, Halestrap AP. The plasma membrane lactate transporter MCT4, but not MCT1, is up-regulated by hypoxia through a HIF-1α-dependent mechanism. J Biol Chem. 2006; 281: 9030–9037. 16452478 10.1074/jbc.M511397200

[32] Bellot G, Garcia-Medina R, Gounon P, Chiche J, Roux D, Pouyssegur J, et al Hypoxia-induced autophagy is mediated through hypoxia-inducible factor induction of BNIP3 and BNIP3L via their BH3 domain. Mol Cell Biol. 2009; 29: 2570–2581. 10.1128/MCB.00166-09 19273585 PMC2682037

[33] Zhang H, Bosch-Marce M, Shimoda LA, Tan YS, Baek JH, Wesley JB, et al Mitochondrial autophagy is an HIF-1-dependent adaptive metabolic response to hypoxia. J Biol Chem. 2008; 283: 10892–10903. 10.1074/jbc.M800102200 18281291 PMC2447655

[34] Yanagihara K, Takigahira M, Tanaka H, Komatsu T, Fukumoto H, Koizumi F, et al Development and biological analysis of peritoneal metastasis mouse models for human scirrhousstomach cancer. Cancer Sci. 2005; 96: 323–332. 15958054 10.1111/j.1349-7006.2005.00054.x PMC11158165

[35] Miyake S, Kitajima Y, Nakamura J, Kai K, Yanagihara K, Tanaka T, et al HIF-1α is a crucial factor in the development of peritoneal dissemination via natural metastatic routes in scirrhous gastric cancer. Int J Oncol. 2013; 43: 1431–1440. 10.3892/ijo.2013.2068 23970191

[36] Yang C, Coker KJ, Kim JK, Mora S, Thurmond DC, Davis AC, et al Syntaxin 4 heterozygous knockout mice develop muscle insulin resistance. J. Clin. Invest. 2001; 107:1311–1318. 11375421 10.1172/JCI12274 PMC209300

[37] Sano H, Eguez L, Teruel MN, Fukuda M, Chuang TD, Chavez JA, et al Rab10, a target of the AS160 Rab GAP, is required for insulin-stimulated translocation of GLUT4 to the adipocyte plasma membrane. Cell Metab. 2007; 5: 293–303. 17403373 10.1016/j.cmet.2007.03.001

[38] Hausdorff SF, Bennett AM, Neel BG, Birnbaum MJ. Different signaling roles of SHPTP2 in insulin-induced GLUT1 expression and GLUT4 translocation. J Biol Chem. 1995; 270: 12965–12968. 7768884 10.1074/jbc.270.22.12965

[39] Micucci C, Orciari S, Catalano A. Hyperglycemia promotes K-Ras-induced lung tumorigenesis through BASCs amplification. PLOS ONE. 2014; 9: e105550 10.1371/journal.pone.0105550 25144301 PMC4140809

[40] Semenza GL. Targeting HIF-1 for cancer therapy. Nat Rev Cancer. 2003; 3: 721–732. 13130303 10.1038/nrc1187

[41] Onnis B, Rapisarda A, Melillo G. Development of HIF-1 inhibitors for cancer therapy. J Cell Mol Med. 2009; 13: 2780–2786. 10.1111/j.1582-4934.2009.00876.x 19674190 PMC2832082

[42] Nagle DG, Zhou YD. Natural product-based inhibitors of hypoxia-inducible factor-1 (HIF-1). Curr Drug Targets. 2006; 7: 355–369. 16515532 10.2174/138945006776054979 PMC2908043

[43] Burroughs S, Kaluz S, Wang D, Wang K, Van Meir EG, Wang B. Hypoxia inducible factor pathway inhibitors as anticancer therapeutics. Future Med Chem. 2013; 5: 553–572. 10.4155/fmc.13.17 23573973 PMC3871878

