# Supplementary figures and images for: The Apoptotic Effect of HIF-1α Inhibition Combined with Glucose plus Insulin Treatment on Gastric Cancer under Hypoxic Conditions
Source: PLoS One. 2015 Sep 4;10(9):e0137257. doi: 10.1371/journal.pone.0137257 (PMC4560388; doi:10.1371/journal.pone.0137257)

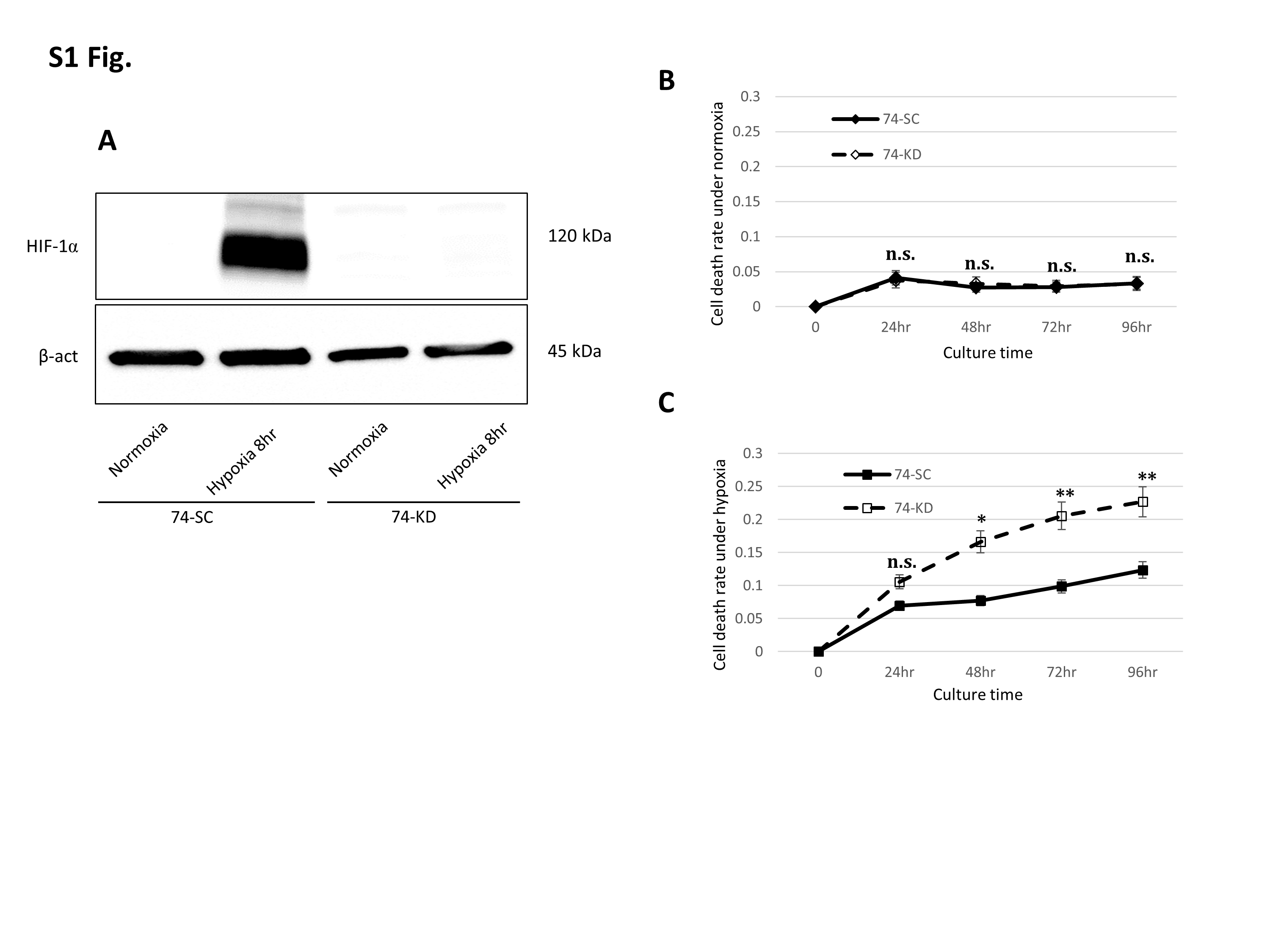

Supplement: S1 Fig — (A) A Western blot analysis of the HIF-1α expression was performed using 74-SC or 74-KD cells under normoxia (20% O2) and hypoxia (1% O2). The HIF-1α expression was completely knocked down in the 74-KD cells after eight hours under hypoxia in comparison to that observed in the 74-SC cells. The internal marker β-actin (β-act) was equally expressed in all cells. (B) The cell death rate was not different between the 74-KD cells and 74-SC cells under normoxia for 24 to 96 hours. (C) The cell death rate in the 74-KD cells was significantly higher than that noted in the 74-SC cells under hypoxia for 48 to 96 hours. n.s.: not significant, *: p<0.05, **: p<0.01, ***: p<0.001 (TIF) [file pone.0137257.s001.TIF]

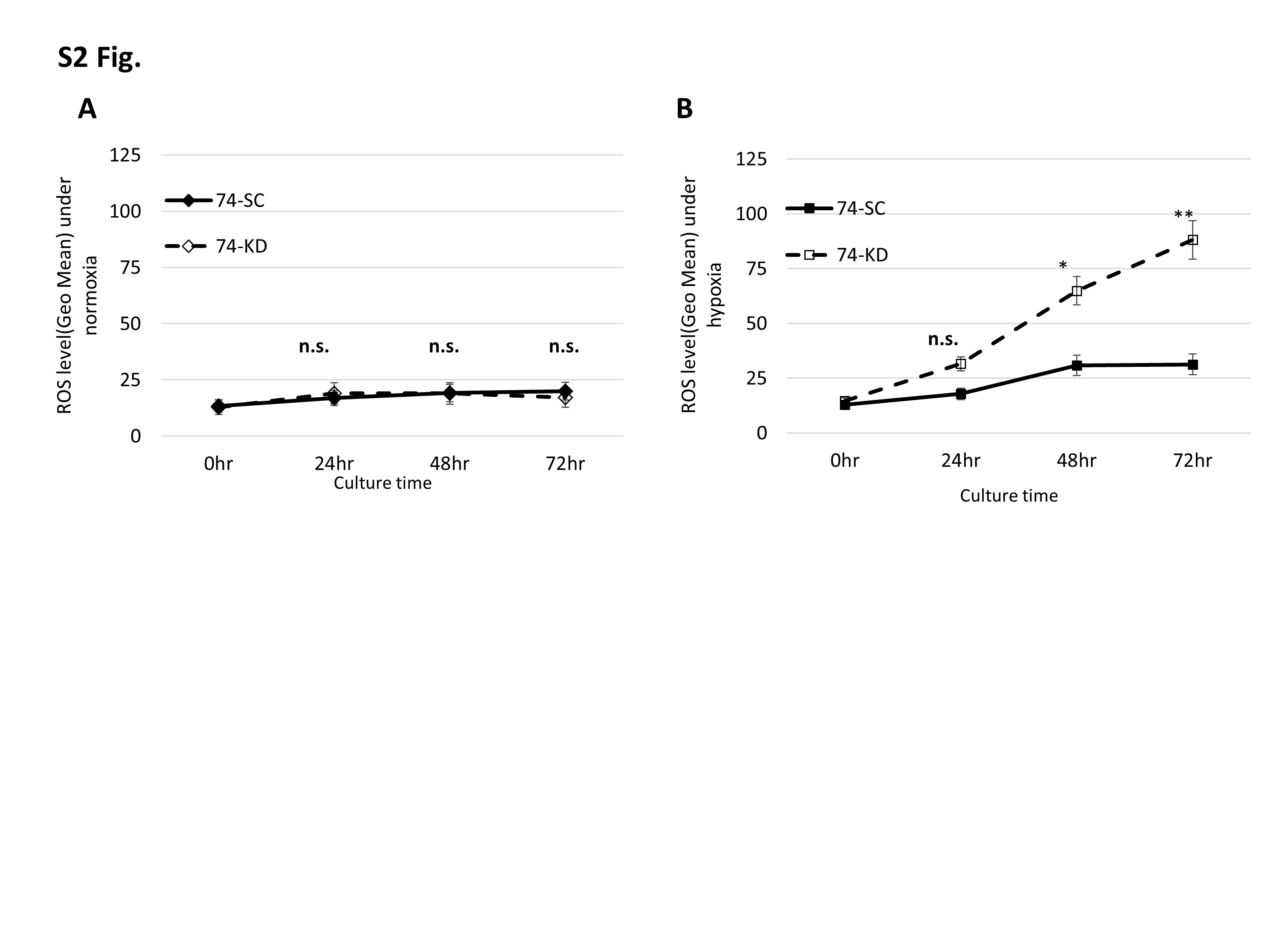

Supplement: S2 Fig — The ROS levels were estimated using 74-SC and 74-KD cells under normoxia (A) and hypoxia (B) for 0 to 72 hours as indicated. n.s.: not significant, *: p<0.05, **: p<0.01, ***: p<0.001 (TIF) [file pone.0137257.s002.TIF]
